# On the Near-Pole Hole Insertion Layer and the Far-Pole Hole Insertion Layer for Multi-Quantum-Well Deep Ultraviolet Light-Emitting Diodes

**DOI:** 10.3390/nano12040629

**Published:** 2022-02-14

**Authors:** Guanting Fang, Min Zhang, Dayuan Xiong

**Affiliations:** 1 Shanghai Key Laboratory of Multidimensional Information Processing, East China Normal University, Shanghai 200241, China; 15957146150@163.com (G.F.); 15071365976@163.com (M.Z.); 2 Key Laboratory of Polarized Materials and Devices (MOE), Department of Electronics, East China Normal University, Shanghai 200241, China

**Keywords:** AlGaN, APSYS, DUV-LED, efficiency droop, hole insertion layer

## Abstract

A novel Multi-Quantum-Well Deep Ultra Violet Light Emitting Diode (DUV-LED) device with a near-pole hole insertion layer and far-pole hole insertion layer was proposed and carefully studied. It was found that remarkable enhancements both in the light output power (LOP) and the internal quantum efficiency (IQE) could be realized by using the far-electrode hole insertion layer and near-electrode hole insertion layer compared to the conventional DUV-LED device. Inserting the near-polar hole insertion layer can increase the electric field in the hole injection layer, which will promote the ionization of the acceptor, increase the hole concentration, and enhance the light-emitting performance of the device. In addition, inserting the far-pole hole insertion layer can suppress electron leakage and promote the hole injection. At the same time, the updated electron barrier height of P-AlGaN/GaN will indirectly weaken the electrostatic field in the hole injection layer, which remains inconducive to the ionization of the acceptor, implying that the electrostatic field between the P-AGaN/GaN layer can optimize the efficiency droop of the device.

## 1. Introduction

Due to the outbreak of the 2019 Corona Virus Disease (COVID-19), methods to effectively prevent and control COVID-19 have quickly become one of the most popular topics. According to related studies, the virus is sensitive to ultraviolet and thermal radiation in the ultraviolet C (UVC) band [1]. Due to this, group III-nitride-based ultraviolet light-emitting diodes represented by AlGaN have aroused great interest. In addition to its effect on the prevention and control of COVID-19, it also has various applications, which encompass water sanitization, disinfection of medicinal apparatus, ultraviolet (UV) therapeutics, solid-state lighting, document validation, phototherapy, medical diagnostics, encrypted communication, and UV interference. Its advantage lies in its energy-saving capacity, small size, high productivity, and long lifetime [2]. The light emission range of the material of AlGaN is adjustable from 200 nm to 365 nm. It is used in various fields by adjusting the emission wavelength [3,4,5]. At all events, despite the fact that AlGaN-based deep ultraviolet LEDs have attracted much attention, some difficult issues are none the less restricting usage of AlGaN-based deep ultraviolet (DUV) LEDs in terms of external quantum efficiency (EQE) and emission power. Degraded internal quantum efficiency (IQE) and inadequate light extraction efficiency (LEE) are the crucial factors leading to such insufficient EQE. Numerous causes have been estimated as being responsible for the low IQE and emission power, for example, the high threading dislocation density (TDD) in AlGaN layers, the spontaneous and piezoelectric polarization, and insufficient activated hole concentration [6]. It is widely believed that the strong electron leakage and poor hole injection may play a critical role in efficiency droop, put down to the large imbalance in electron and hole injection in AlGaN-based DUV-LEDs and, subsequently, reduced radiative recombination efficiency. In addition to its unique band-engineered structure, a different structural parameter optimization can effectively manage carrier confinement phenomena properly [7]. Prior to this paper, many different band-engineering structures were proposed, for example the optimization of the structure of the last quantum barrier [8,9,10], the optimization of the structure of the electron blocking layer (EBL) [11,12,13], and the design of the insertion layer between the active region and the electron blocking layer [14,15]. However, there are a limited number of studies available, which focus on the hole injection layer, for instance the superlattice structure in the hole injection layer [16,17], and insertion of the p-AlGaN/n-AlGaN/p-AlGaN current spreading layer into the hole injection layer [18]. It is rarely considered that the piezoelectric polarization field between the hole injection layer and the contact layer affects the performance of the DUV-LED.

In this paper, we put forward the concepts of far-pole hole insertion layer and near-pole hole insertion layer for the Multi-Quantum-Well DUV-LED. The far-pole hole insertion layer can increase the maximum IQE, but the efficiency drop will be more obvious, while the near-pole hole insertion layer slightly reduces the maximum IQE, but the efficiency declines slowly. Inserting these two insertion layers on the traditional structure not only increases the maximum IQE, but also reduces the efficiency drop rate. The results suggest that the electrostatic field of contact layer could alleviate the efficiency droop for the Multi-Quantum-Well DUV-LED device.

## 2. Structure and Parameter

**Figure 1:**
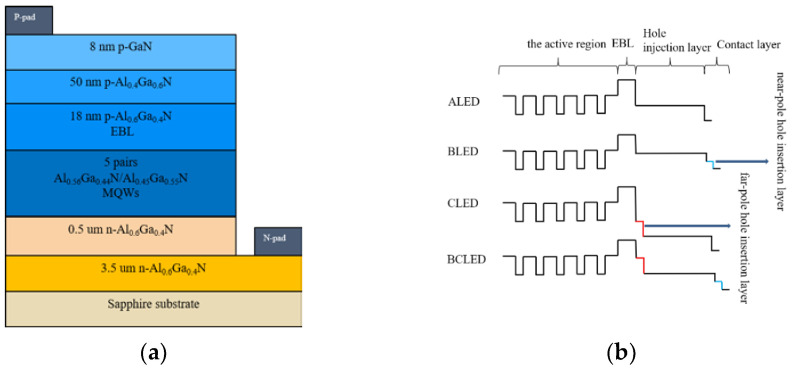
(**a**) Schematic diagram of traditional structure ALED. (**b**) Schematic conduction band profiles of the four structures (ALED, BLED, CLED, BCLED).

The traditional AlGaN-based DUV-LED structure, deemed as the reference (labeled as ALED), is usually grown on a *c*-plane sapphire substrate. The conventional AlGaN-based DUV-LED structure reported by Hirayama et al. [19] have been reoptimized and used as a reference. The rest of the structure includes a 4-μm-thick n-type Al_0.60_Ga_0.40_N layer, followed by a five-period multiple-quantum-well (MQW) consisting of 3-nm-thick Al_0.45_Ga_0.55_N wells and 12-nm-thick Al_0.56_Ga_0.44_N barriers, next, a 18-nm-thick Mg-doped Al_0.60_Ga_0.40_N EBL, a 50-nm-thick p-type Al_0.40_Ga_0.60_N hole injection layer, and an 8-nm-thick Mg-doped GaN contact layer over the structure. A schematic diagram of the traditional structure ALED is shown in Figure 1a. Based on the reference structure (i.e., ALED), three modified structures are proposed and compared critically (labeled as BLED, CLED and BCLED). The structure of BLED is identical to ALED except for near-pole hole insertion layer, which inserts a 5-nm-thick Mg-doped Al_0.35_Ga_0.65_N insertion layer (labeled as the near-pole hole layer) between the Al_0.40_Ga_0.60_N hole injection layer and p-GaN contact layer. The structure of CLED, is based on the traditional structure ALED with a 5-nm-thick Mg-doped Al0.45Ga0.55N far-pole hole insertion layer, which is inserted between the hole injection layer and electron blocking layer (EBL). The structure BCLED is based on the structure ALED in which the near-pole hole insertion layer and the far-pole hole insertion layer are inserted. Figure 1b shows the schematic conduction band profiles of structures ALED, BLED, CLED and BCLED.

In this research, the four structures (ALED, BLED, CLED, BCLED) are studied using the Advanced Physical Model of Semiconductor Devices (APSYS) simulation software [20]. In order to ensure that the simulation conforms to the actual experiment, the band-offset ratio, defined by the ratio between the conduction band offset and the valence band offset, is set at 50:50 [21]. The Shockley–Read–Hall (SRH) recombination lifetime and the Auger recombination coefficient are severally set to 10 ns [22] and 1.0 × 10^−30^ cm^6^/s [23]. According to the method proposed by Fiorentini et al. [24], due to the spontaneous polarization and piezoelectric polarization of the AlGaN/AlGaN heterojunction, polarization charges will appear at the interface. Taking into account material defects, the interface charge density is 40% [25,26]. The light extraction efficiency is set to ~8% for DUV-LEDs [27]. In addition, other material parameters adopted in our simulation can be found in research by [28].

## 3. Results and Discussion

**Figure 2:**
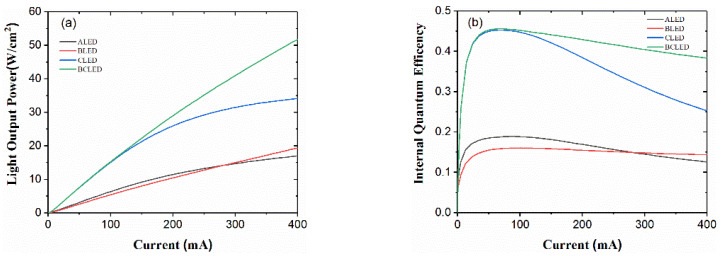
(**a**) Comparison of output power and (**b**) IQE plots of ALED, BLED, CLED and BCLED with respect to the current density.

In order to reflect the supremacy of the BLED, CLED and BCLED design over the traditional structure ALED, the performance of the devices is studied in terms of IQE and LOP at different injection current densities as shown in Figure 2a,b. Table 1 lists the performance comparison of the output parameters. As the current increases, the light output power of the LED increases sharply. In addition, compared with ALED, the light output power in the case of CLED and BCLED increases faster. A 3.2-fold higher light output power compared with ALED is viewed in BCLED at a current of 400 mA. Similarly, the efficiency droop is lowered by 16% in the BCLED with enhanced maximum IQE with reference to ALED. A 1.13-fold higher light output power compared with ALED is viewed in BLED at a current of 400 mA. Notably, the efficiency droop is only lowered by 10% in the BLED. In terms of the internal quantum efficiency of 400 mA, the BCLED structure is preferred. The characteristic curve of efficiency droop at a current of 400 mA bolster the supremacy of BLED structure.

**Figure 3:**
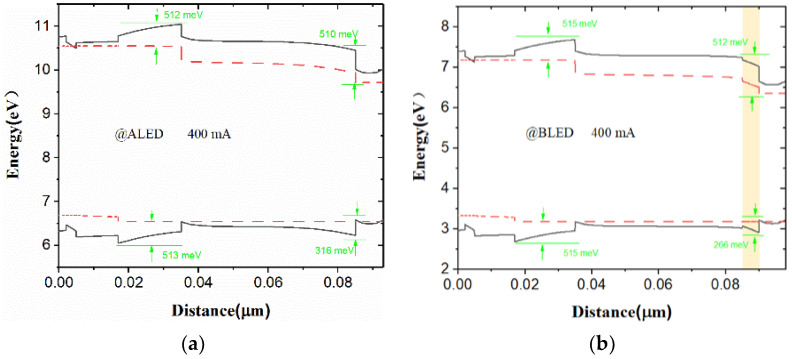

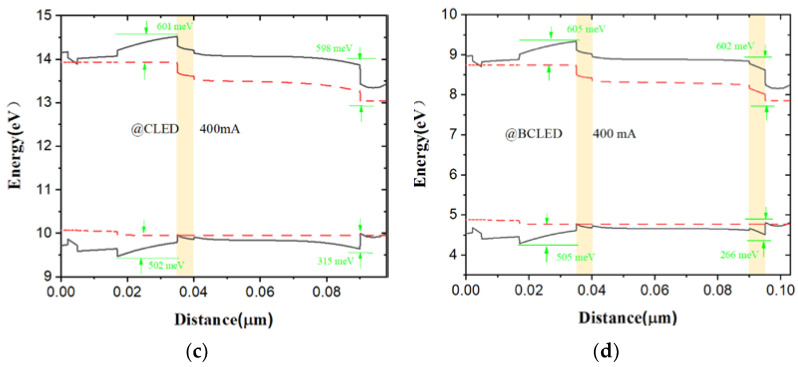
Energy band diagrams of (**a**) ALED, (**b**) BLED, (**c**) CLED, (**d**) BCLED at 400 mA respectively.

Figure 3 shows the energy band diagrams (black solid lines) and quasi fermi level (red dashed lines) of the four structures at the injection current of 400 mA, respectively. The energy difference between band edge (conduction and valance band) and quasi fermi level of the carrier is defined as the barrier potential height for carrier (electron and hole). Electron blocking layer (EBL) plays a vital role for better confinement of electron and injection of hole. The contact layer has a certain effect on the rate of efficiency drop. From Figure 3a,b it can be inferred that for BLED the hole potential barrier height at the p-AlGaN/GaN interface decreases from 316 meV to 265 meV contrasted to that of ALED. As the hole barrier height at the p-AlGaN/GaN interface decreases, it slows down the efficiency droop, which makes BLED perform better than ALED. In addition, from Figure 3a,c it can be deduced that for CLED the potential barrier height, compared to ALED, increases from 512 meV to 601 meV for the electrons. Compared with ALED, the electron barrier height of the electron blocking layer is increased by 89 meV to reduce the leakage of electrons, and thus ensuring that the performance of CLED is better than that of ALED. Moreover, it is because the electron barrier height of the electron barrier layer has increased by 89 meV, that has caused the electron barrier height at the p-AlGaN/GaN interface of the CLED to increase from 510 meV to 598 meV. Consequently, the electronic barrier height at the p-AlGaN/GaN interface has increased by 88 meV, thereby accelerating the efficiency decline. Therefore, the LOP of CLED rises slowly under high current injection compared to ALED. The effective barrier height of the valence band of CLED is significantly lower than that of ALED, leading to the improved efficiency of the hole injection into from P-type region to the active region. BCLED is a combination of BLED and CLED, and its energy band diagram characteristics have both the advantages of BLED and CLED.

**Figure 4:**
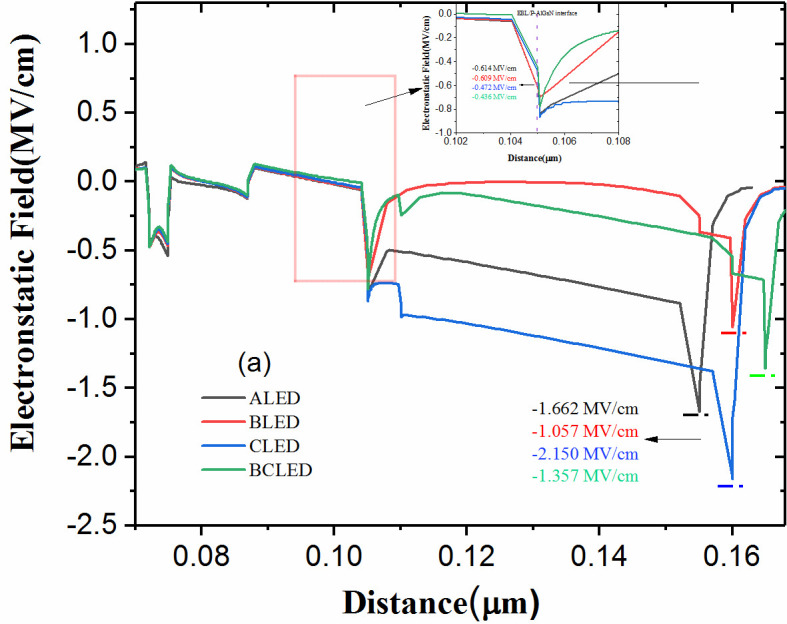

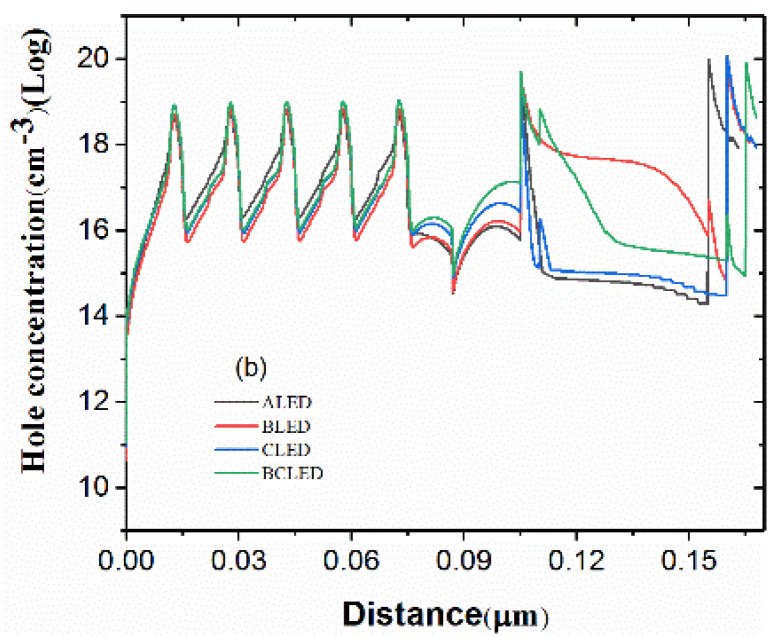
(**a**) Electrostatic field and (**b**) hole concentrations of the four structures at 400 mA.

Considering that the electric difference field is mainly on both sides of the hole injection layer, as a result, the electrostatic fields of structures A–D near the P-AlGaN layer under the injected current of 400 mA are plotted in Figure 4. The values of the electrostatic field at the EBL/p-AlGaN interface are −0.614, −0.609, −0.472 and −0.436 MV/cm, for structures A–D, and the electrostatic field at the p-AlGaN/p-GaN interface are −1.662, −1.057, −2.150 and −1.357 mV/cm, respectively. These data indicate that the polarization induced electrostatic field at p-AlGaN/p-GaN interface can be effectively reduced by using the near-pole hole insertion layer structures. In particular, the downward bending of the p-AlGaN/p-GaN interface affects the electrostatic field of the hole injection layer. It also shows that by using a far-pole hole insertion layer, the polarization-induced electrostatic field at the EBL/p-AlGaN interface can be effectively reduced to mitigate electron leakage. Compared with the traditional structure of ALED, the more downward band bending at the p-AlGaN/p-GaN interface increases the electron effective barrier height here and accelerates the efficiency droop.

**Figure 5:**
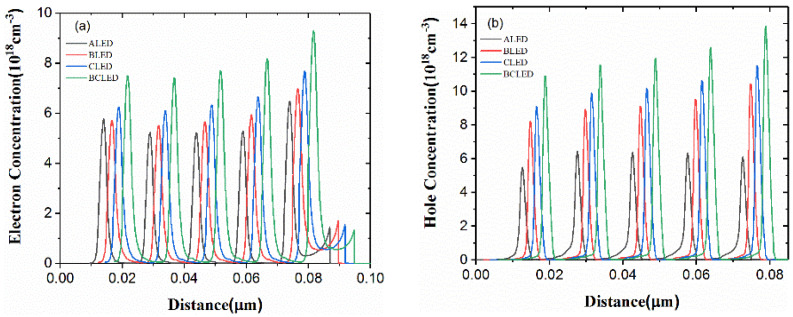
(**a**) Electron concentrations and (**b**) hole concentrations of the four structures at 400 mA.

Figure 5 shows the electron and hole concentration distributions of the four structures in the active region. It is noteworthy that the horizontal position of four structures has been shifted slightly for better observation. It shows that CLED with the far-electrode hole insertion layer achieved a higher carrier concentration in MQWs, compared with ALED. More effective electron blocking and higher hole injection efficiency lead to this result. It shows that BLED with a near-electrode hole insertion layer achieved higher carrier concentration in MQWs, compared with ALED. Compared with ALED, although BLED’s hole injection efficiency remains unchanged, more holes will be injected into the active region as more holes are ionized in the hole injection layer.

**Figure 6:**
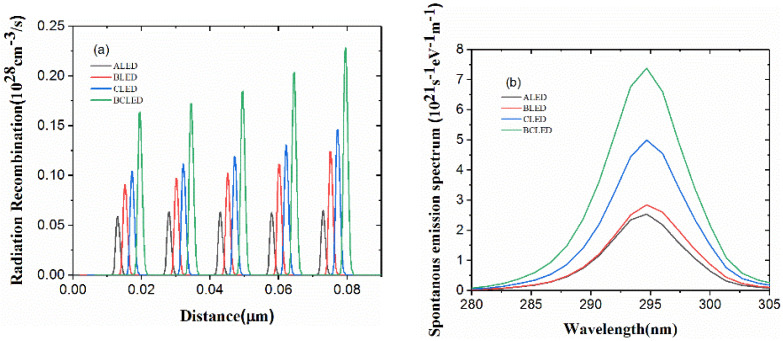
(**a**) Spontaneous emission spectrum and (**b**) radiative recombination rates at 400 mA.

The effective radiation recombination rate of four different structures is shown in Figure 6a. Compared with ALED, the rations of improved recombination rates for BLED, CLED and BCLED are 52%, 76% and 170%, respectively. This can be owed to the superior carrier concentration as shown above. Figure 6b shows the spontaneous emission spectra of these four structures at 400 mA. The law of radiation recombination rate of the four structures can also be reflected in the emission spectrum. The intensity of the emission spectrum of the structure is proportional to the radiation recombination rate. It indicates that the peak emission wavelength is almost identical in both structures and occurs at 294.7 nm. It is easy to see that BLED, CLED and BCLED clearly improves the light output property of DUV-LED. The spectral peak of BLED, CLED and BCLED increased severally by 12.17%, 97.31% and 191.25% compared with the conventional ALED.

## 4. Conclusions

An AlGaN-based Multi-Quantum-Well DUV-LED with far-electrode hole insertion layer and near-electrode hole insertion layer was proposed and investigated. It was found that remarkable enhancements both in the LOP and IQE could be realized by employing a far-electrode hole insertion layer and near-electrode hole insertion layer compared with the conventional AlGaN-based DUV-LEDs. Inserting the near-polar hole insertion layer can increase the electric field in the hole injection layer, which promotes the ionization of the acceptor, increases the hole concentration, and enhances the light-emitting performance of the device. In addition, inserting the far-pole hole insertion layer can suppress the electron leakage and promote the hole injection. At the same time, the updated electron barrier height of P-AlGaN/GaN will indirectly weaken the electrostatic field in the hole injection layer, which remains inconducive to the ionization of the acceptor, suggesting that the electro-static field between the P-AGaN/GaN layer can optimize the efficiency droop of the device. This work provides a new idea for slowing down the IQE of the AlGaN-based Multi-Quantum-Well DUV-LED device.

## Figures and Tables

**Table 1:** Comparison between Maximum IQE, Efficiency Droop, and Light Output Power of Different Structures.

Structure	Maximum IQE (%)	IQE Droop @400 mA (%)	LOP (W/cm^2^)
ALED	18.9	33.3	16.4
BLED	16.0	10.0	18.5
CLED	45.3	44.2	33.3
BCLED	45.6	16	52.7

## Data Availability

The data presented in this study are available on request from the corresponding author.
